# STAT3 isoform dynamics reveal robust splice ratio maintenance across cytokine-activated human immune cells

**DOI:** 10.3389/fimmu.2026.1792173

**Published:** 2026-04-01

**Authors:** Nils Ott, Bodo Grimbacher, Virginia Andreani

**Affiliations:** 1 Institute for Immunodeficiency, Center for Chronic Immunodeficiency (CCI), Medical Center, Faculty of Medicine, University of Freiburg, Freiburg im Breisgau, Germany; 2 Department of Rheumatology and Clinical Immunology, University Medical Center Freiburg, Freiburg im Breisgau, Germany; 3 Center for Integrative Biological Signaling Studies (CIBSS), University of Freiburg, Freiburg im Breisgau, Germany; 4 Resolving Infection Susceptibility (RESIST) - Cluster of Excellence 2155 to Hannover Medical School, Satellite Center Freiburg, Freiburg im Breisgau, Germany; 5 German Center for Infection Research (DZFI), Satellite Center Freiburg, Freiburg im Breisgau, Germany

**Keywords:** alternative splicing, hyper-IgE syndrome, isoform expression, JAK-STAT signaling, splice ratio, STAT3 isoforms, STAT3α, STAT3β

## Abstract

Alternative splicing of STAT3 produces two principal isoforms, STAT3α and STAT3β, that differ in transactivation capacity and DNA-binding behavior. Whereas STAT3α is thought to be a transcriptional activator, STAT3β – due to the lack of the transactivation domain – is thought to be a transcriptional repressor. Therefore, the relative abundance of STAT3α and STAT3β molecules within a leukocyte will be critical for immune homeostasis and for gene-therapeutic strategies targeting STAT3. This study investigates whether short-term exposure to IFN-α, IL-6/sIL-6Rα, or IL-10 alters STAT3α/STAT3β stoichiometry in purified human CD4^+^, CD8^+^, CD14^+^ and CD19^+^ cells. We evaluated in healthy donors PBMCs the STAT3α and STAT3β mRNA and protein levels after stimulation with IFN-α, IL-6/sIL-6Rα or IL-10. For mRNA analysis, twelve candidate reference genes were assessed for stability across subsets and stimuli, with *UBE2D2* identified as the most stable reference gene. We demonstrate that at mRNA level, cytokine treatment induced *STAT3α* and *STAT3β* mRNA in a subset-dependent manner. *STAT3β* mRNA largely paralleled *STAT3α*, and crucially, the *STAT3α*/*STAT3β* mRNA ratio remained constrained within a narrow range. At protein level, STAT3α predominated markedly, yielding a pronounced transcript-protein decoupling. Together, these data indicate that *ex vivo* short-term cytokine signaling with IFN-α, IL-6/sIL-6Rα, and IL-10 co-induces STAT3 isoforms without broadly reprogramming their splice balance in primary human leukocytes and underscore the importance of preserving physiological isoform ratios in gene-therapeutic strategies targeting STAT3. These findings suggest that short-term inflammatory signals alone are insufficient to shift STAT3 isoform production, supporting the idea that additional or more prolonged regulatory inputs are required to modulate STAT3 splicing *in vivo* and that physiologically, the STAT3α/STAT3β ratio may be modulated at protein level.

## 1 Introduction

Cytokine responses are executed through tightly regulated signaling cascades that translate extracellular cues into lineage- and context-specific transcriptional programs. The Janus kinase (JAK)-signal transducer and activator of transcription (STAT) pathway is a central conduit for these signals: ligand binding triggers receptor/JAK activation, phosphorylation of STATs, dimerization, nuclear translocation, and target gene regulation (1, 2). STAT3 is a multifunctional transcription factor activated by multiple cytokines including members of the IL-6 and IL-10 family or type I interferons, and implicated in development, immune homeostasis, and inflammation (3–5).

The human *STAT3* locus generates multiple protein isoforms, primarily STAT3α and STAT3β, through alternative splicing and proteolytic processing. STAT3α (~92 kDa) contains the full C-terminal transactivation domain (TAD) and the S727 phosphorylation site, whereas STAT3β (∼83 kDa) lacks most of the TAD, carries a unique FIDAVWK C-terminus, and lacks S727 (6). STAT3β differs from STAT3α in DNA binding, dimer stability and nuclear retention, and exhibits both overlapping and unique transcriptional outputs (7–10). *In vivo*, loss of STAT3β produces phenotypes distinct from loss of STAT3α, supporting non-redundant functions and the biological relevance of isoform stoichiometry (9–12). Dynamic regulation of the STAT3α/STAT3β ratio has been implicated in myeloid maturation and inflammatory responses (12–15), and recent work identified poly-C binding protein 1 (PCBP1) as a key regulator capable of shifting splicing toward STAT3β (16). However, the upstream stimuli that modulate PCBP1 activity remain poorly defined, leaving unresolved which and how physiological stimuli influence STAT3 splicing.

Isoform balance may be clinically relevant for monogenic diseases affecting *STAT3*, such as the dominant-negative STAT3 hyper-IgE syndrome, or the STAT3 gain-of-function type of common variable immunodeficiency (17–19). How canonical STAT3-engaging cytokines acutely affect STAT3α and STAT3β isoform expression across primary human leukocyte subsets has not been studied so far. This question, however, is of critical importance while designing gene therapy approaches for these monogenic conditions.

In this study, we evaluated STAT3 isoform balance in a cell-type-specific manner following acute cytokine exposure. We therefore established and validated isoform-specific qRT-PCR assays, performed a reference gene assessment and quantified STAT3α and STAT3β expression at mRNA and protein level in purified human CD4^+^ and CD8^+^ T cells, CD14^+^ monocytes and CD19^+^ B cells following acute exposure to IFN-α, IL-6/sIL-6Rα and IL-10. Our aim was to determine whether short-term cytokine stimulation alters the STAT3 isoform stoichiometry in a cell-type-specific manner and to discuss its implications for gene-therapeutic strategies in STAT3-associated diseases.

## 2 Materials and methods

### 2.1 Human material and ethics approval statement

Anonymized human leukocyte concentrates from eight independent healthy adult blood donors were obtained from the Institute for Transfusion Medicine and Gene Therapy, Medical Center, University of Freiburg, and processed according to institutional guidelines. All experiments used de-identified material and were performed under approval by the local ethics committee and in accordance with applicable regulations (ethics protocol number 282/11, 302/13 and 354/19). Samples were collected with the written consent of all study participants at the University of Freiburg.

### 2.2 PBMC isolation, sorting and stimulation

Peripheral Blood Mononuclear Cells (PBMCs) were isolated by density gradient centrifugation (Pancoll (PAN-Biotech # P04-601000), SepMate™) from EDTA blood diluted 1:2 in FACS buffer (2% FBS in PBS). Cells were washed, subjected to erythrocyte lysis, counted on a Neubauer hemocytometer, and processed immediately. PBMCs were surface-stained with a mix of fluorochrome-conjugated antibodies (CD4-APC-Cy7 (Biolegend #300518), CD8-PerCP-Cy5.5 (Invitrogen #45-0088-42), CD14-PE (BD Biosciences #555398), CD19-BV421 (Biolegend #302234)) and subsets were purified by fluorescence-activated cell sorting (MoFlo Astrios EQ or FACSAria Fusion). Gates selected were CD14^+^CD19^-^ (monocytes), CD19^+^CD14^-^ (B cells) and CD14^-^CD19^-^ CD4^+^ or CD8^+^ (T cells). Purities exceeded standard sorting thresholds. Sorted cells were incubated with RPMI and treated immediately with recombinant human cytokines at 100 ng/ml: IFN-α (Abcam #ab48750), IL-6/sIL-6Rα (Peprotech #200–06 and #200-06RC), or IL-10 (Peprotech #200-10) for 6 h (mRNA analysis) or 8 h or 16 h (for protein analysis). Untreated controls received RPMI only. After stimulation, ice-cold PBS was added, and reaction plates were placed on ice to stop the stimulation. Cells were harvested to isolate RNA or protein as described.

### 2.3 Genomic DNA extraction, RNA extraction and cDNA synthesis

Genomic DNA and total RNA were isolated using the QIAamp DNA Mini Kit (Qiagen) and RNeasy^®^ Mini Kit (Qiagen) including on-column DNase digestion, respectively, following the manufacturer´s protocols. Equipment and surfaces were cleaned with RNase AWAY^®^ and only filter pipette tips were used. RNA concentration and purity were measured by NanoDrop™ 2000 spectrophotometer. RNA was reverse-transcribed into cDNA using M-MLV Reverse Transcriptase (Promega #M1701), according to the supplier’s instructions.

### 2.4 Isoform specific primers, qRT-PCR and reference gene evaluation

Isoform-specific primers targeted the exon-22/23 region: a common forward primer in exon 21 and isoform-specific reverse primers spanning the exon junction, yielding 136 bp (*STAT3α*) and 140 bp (*STAT3β*) products. Primer specificity was confirmed by PCR, gel electrophoresis and no-reverse-transcriptase controls. PCR efficiency was determined from ten-fold cDNA dilutions. qRT-PCR was performed in triplicates using SYBR^®^ Green reagent (Thermo Fisher Scientific #4309155) on a LightCycler^®^ 480 II PCR system (Roche Diagnostics). Reference gene stability ranking used RefFinder (geNorm, NormFinder, BestKeeper, comparative ΔC_q_). *STAT3* mRNA relative expression to *UBE2D2* was calculated with the 2^-ΔΔCq^ method.

### 2.5 Immunoblot analysis

Whole cell lysates of sorted CD4^+^ and CD8^+^ cells were obtained by incubating the cells with lysis buffer (50 mM TRIS pH 8.0, 150 mM NaCl, 1% Triton X-100, 0.1% SDS) and protease inhibitor mix (Roche #05892970001), for 20 min on ice. Lysates were centrifuged at 16000 rpm for 15 min at 4 °C. Protein concentration of the supernatant was determined by BCA assay. Samples with equal amounts of protein were denatured, separated on 8% SDS-PAGE, and transferred to PVDF membranes. Membranes were blocked with 5% milk/TBS-T and probed overnight with an antibody to the N-terminal part of the STAT3 protein (Cell Signaling #9139) that allows isoform discrimination (STAT3α (86 kDa) and STAT3β (79 kDa)); HRP-conjugated secondary antibody (Santa Cruz #SC516102) was used. Immunoblots were developed with Fusion Fx (Vilber Lourmat) and analyzed with Image Studio Light 5.2 (LI-COR Biosciences). Values were normalized to a loading control (Tubulin, Proteintech #HRP-66031). At least three biological replicates were analyzed.

### 2.6 Statistics

Data are expressed as mean ± SD from the indicated number of independent donors. Data were analyzed by two-way ANOVA with Tukey’s multiple comparisons for multi-group analyses, including a Mixed-effects analysis, using the GraphPad Prism program. To account for inter-donor variability, experiments were carried out independently for each donor and stimulation conditions were analyzed in a donor-paired manner, with statistical testing performed on paired data. P values of less than 0.05, 0.01, and 0.001 were considered significant.

## 3 Results

### 3.1 Design and validation of *STAT3α*- and *STAT3β*-specific qRT-PCR assays

**Figure 1:**
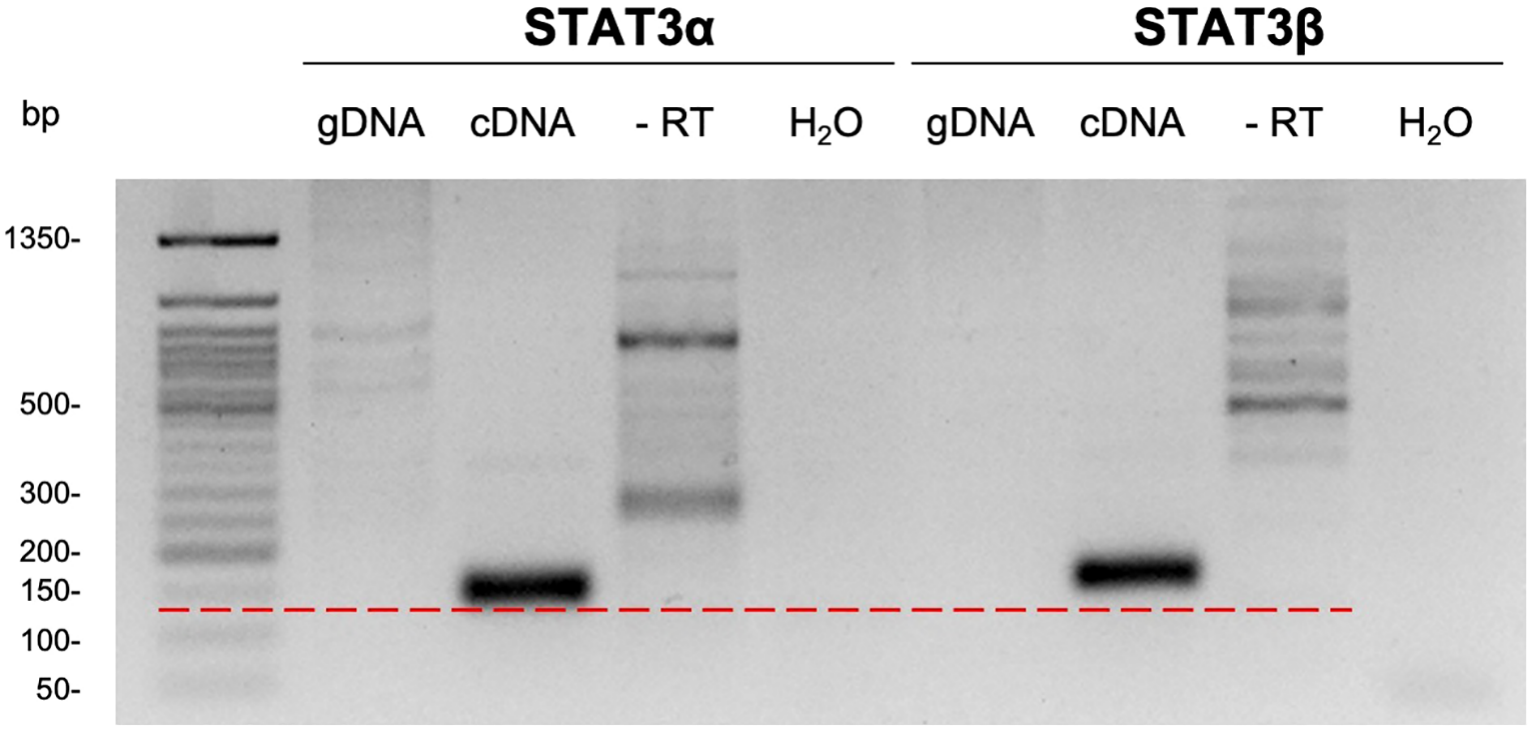
Isoform-specific amplification of *STAT3α* and *STAT3β* mRNA in PCR reaction. *STAT3α* and *STAT3β* specific forward and reverse primers were used in four PCR reactions with different DNA templates. Either genomic DNA (gDNA, lane 2 and 6) of T cells, cDNA synthesized with or without a reverse transcriptase (RT) from RNA of T cells (cDNA, lane 3 and 7; -RT, lane 4 and 8) or no DNA template (H_2_O, lane 5 and 9) were used. PCR reactions were carried out with different annealing temperatures, here 64 °C is shown. After the amplification DNA samples were run on a 2.5% agarose gel for 90 minutes. The red dotted line indicates the amplicon size of approximately 130 bp that is tangent to the *STAT3α* cDNA band (lane 3) but is not touched by the *STAT3β* cDNA band (lane 7).

To enable accurate quantification of *STAT3α* and *STAT3β* transcripts in primary human lymphocytes, isoform-specific qRT-PCR assays were designed and validated. A common forward primer positioned in exon 21 (5′-TGACATTCCCAAGGAGGAGG-3′) and isoform-specific reverse primers spanning the exon 22–23 junction (STAT3α_rv: 5′-ATTGCTGCAGGTCGTTGGTG-3′, STAT3β_rv: 5′-TCCAAACTGCATCAATGAATGGTG-3′) generated amplicons with expected lengths of 136 bp (*STAT3α*) and 140 bp (*STAT3β*). Annealing-temperature optimization by gradient PCR identified 64 °C as optimal, producing a single product of the expected size for each isoform and no specific amplification in no-reverse-transcriptase or no-template controls (**Figure 1**; **Supplementary Figure 1**). Genomic DNA controls confirmed cDNA specificity. Serial dilution curves demonstrated reaction efficiencies of 107.81% for *STAT3α* and 104.61% for *STAT3β*, meeting typical quality criteria for comparative qPCR and indicating robust amplification characteristics across a broad dynamic range. These results demonstrate selective and efficient amplification of the *STAT3α* and *STAT3β* cDNA targets suitable for quantitative expression analysis in primary immune cells.

### 3.2 *UBE2D2* is the most reliable reference gene in lymphocytes for *STAT3α* and *STAT3β* mRNA evaluation

**Figure 2:**
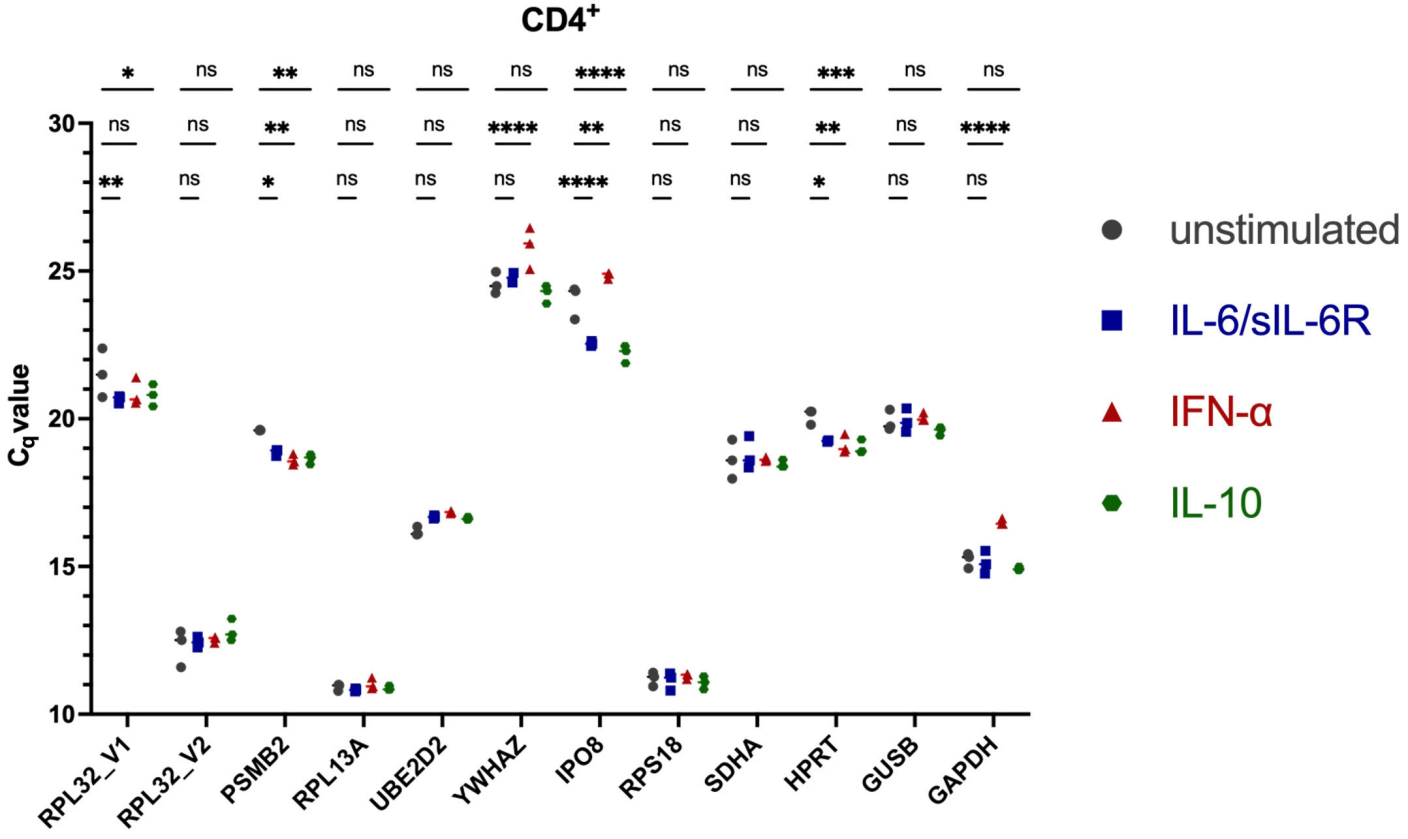
Real-time qRT-PCR of 12 candidate reference genes in untreated and stimulated isolated CD4^+^ PBMCs. Shown are the individual threshold cycle (C_q_) values of the candidate reference genes in unstimulated and stimulated CD4^+^ cells. PBMCs were isolated by density gradient separation, sorted by fluorescence-activated cell sorting (FACS), and stimulated without (grey symbols) or with IL-6/IL-6Rα (100 ng/ml, blue symbols), IFN-α (100 ng/ml, red symbols) or IL-10 (100 ng/ml, green symbols) for 300 min. After RNA extraction and cDNA synthesis, C_q_ values were determined *via* RT-qPCR. Differences in C_t_ values between unstimulated and stimulated cells were tested in three experiments for statistical significance by Two-way ANOVA with Tukey’s multiple comparisons test. The mean and individual values are shown (***p < 0.001; **p < 0.01; *p < 0.05; and ns, non-significant).

**Figure 3:**
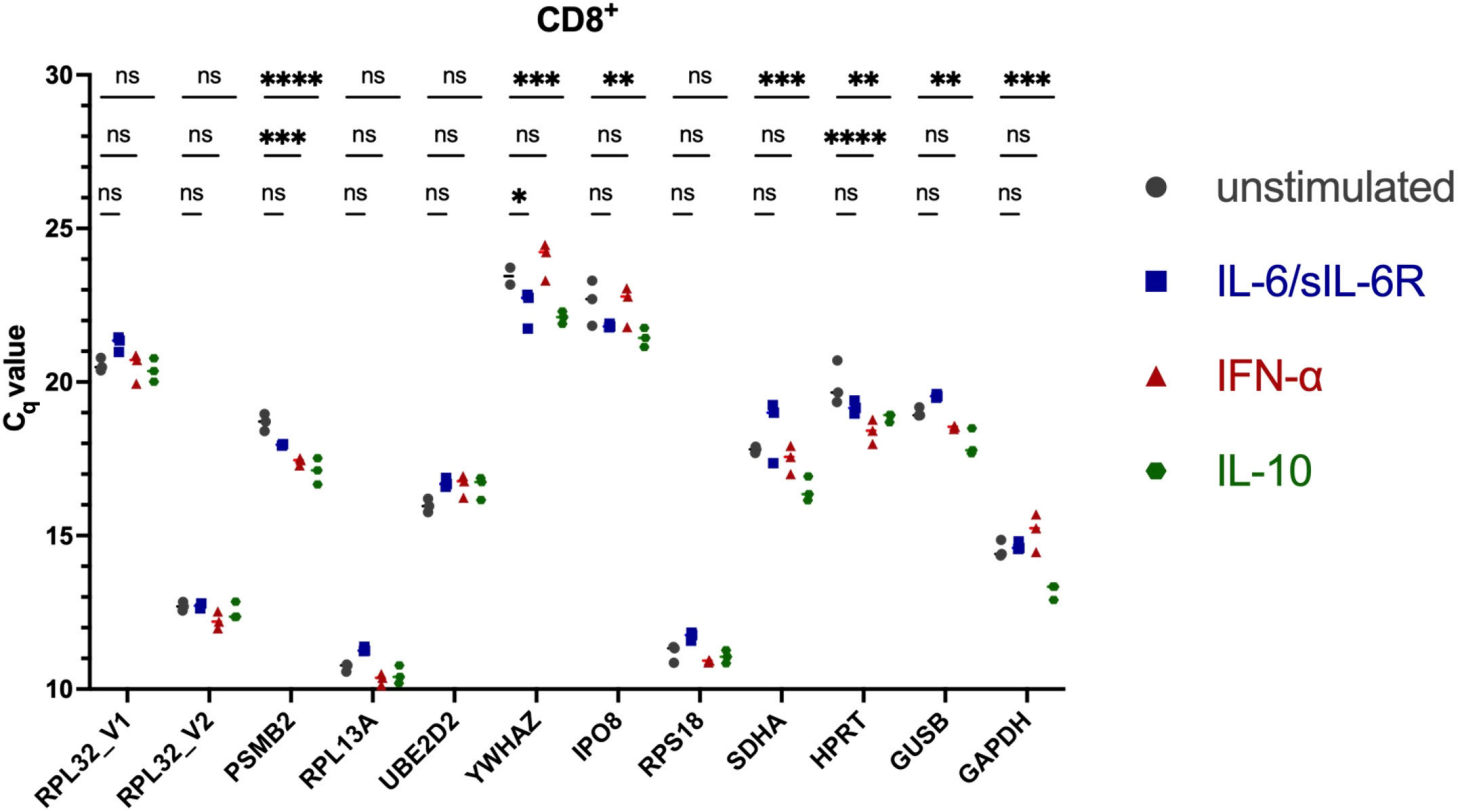
Real-time qRT-PCR of 12 candidate reference genes in untreated and stimulated isolated CD8^+^ PBMCs. Shown are the individual threshold cycle (C_q_) values of the candidate reference genes in unstimulated and stimulated CD8^+^. PBMCs were isolated by density gradient separation, sorted by fluorescence-activated cell sorting (FACS), and stimulated without (grey symbols) or with IL-6/IL-6Rα (100 ng/ml, blue symbols), IFN-α (100 ng/ml, red symbols) or IL-10 (100 ng/ml, green symbols) for 300 min. After RNA extraction and cDNA synthesis, C_q_ values were determined *via* RT-qPCR. Differences in C_t_ values between unstimulated and stimulated cells were tested in three experiments for statistical significance by Two-way ANOVA with Tukey’s multiple comparisons test. The mean and individual values are shown (***p < 0.001; **p < 0.01; *p < 0.05; and ns, non-significant).

**Figure 4:**
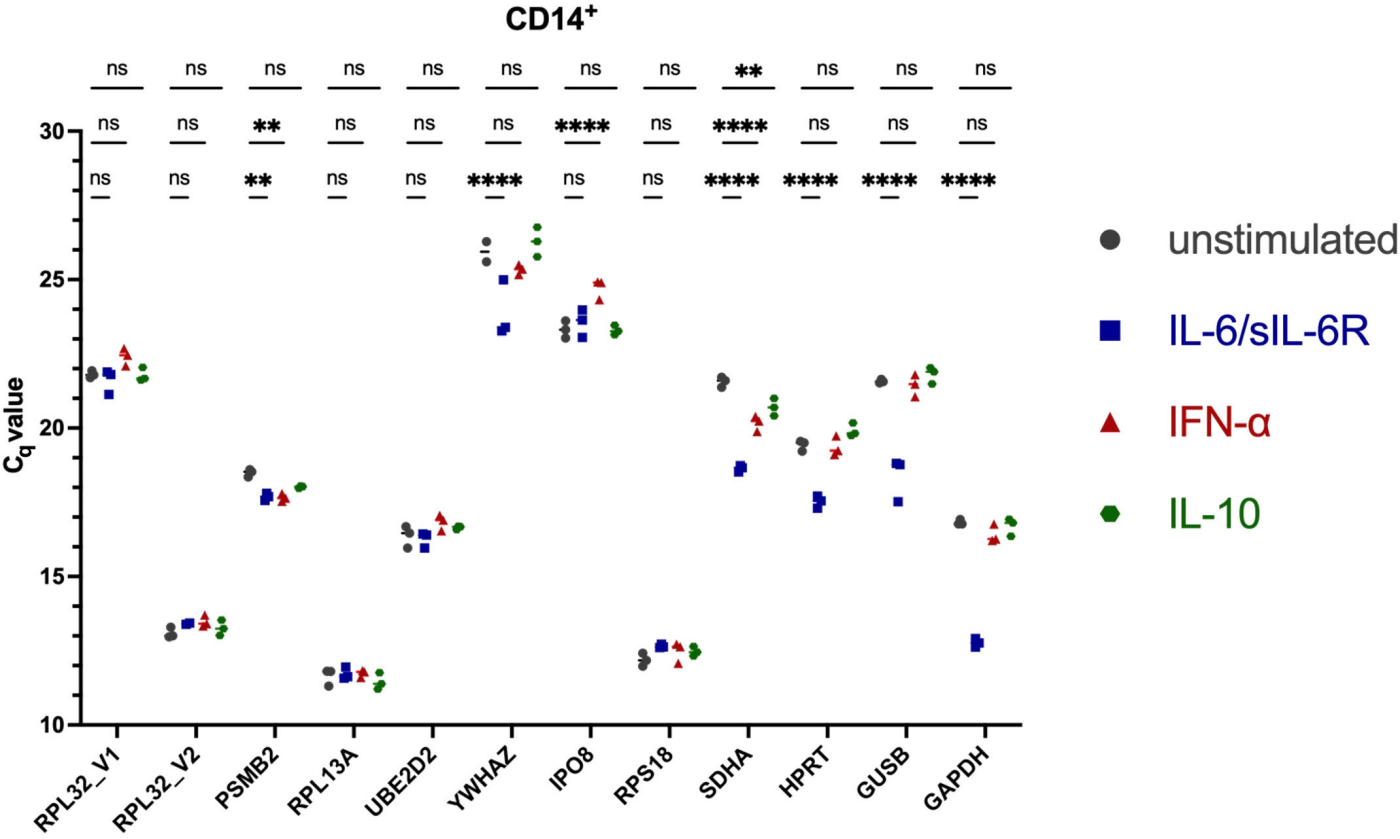
Real-time qRT-PCR of 12 candidate reference genes in untreated and stimulated isolated CD14^+^ PBMCs. Shown are the individual threshold cycle (C_q_) values of the candidate reference genes in unstimulated and stimulated CD14^+^ cells. PBMCs were isolated by density gradient separation, sorted by fluorescence-activated cell sorting (FACS), and stimulated without (grey symbols) or with IL-6/IL-6Rα (100 ng/ml, blue symbols), IFN-α (100 ng/ml, red symbols) or IL-10 (100 ng/ml, green symbols) for 300 min. After RNA extraction and cDNA synthesis, C_q_ values were determined *via* RT-qPCR. Differences in C_t_ values between unstimulated and stimulated cells were tested in three experiments for statistical significance by Two-way ANOVA with Tukey’s multiple comparisons test. The mean and individual values are shown (***p < 0.001; **p < 0.01; *p < 0.05; and ns, non-significant).

**Figure 5:**
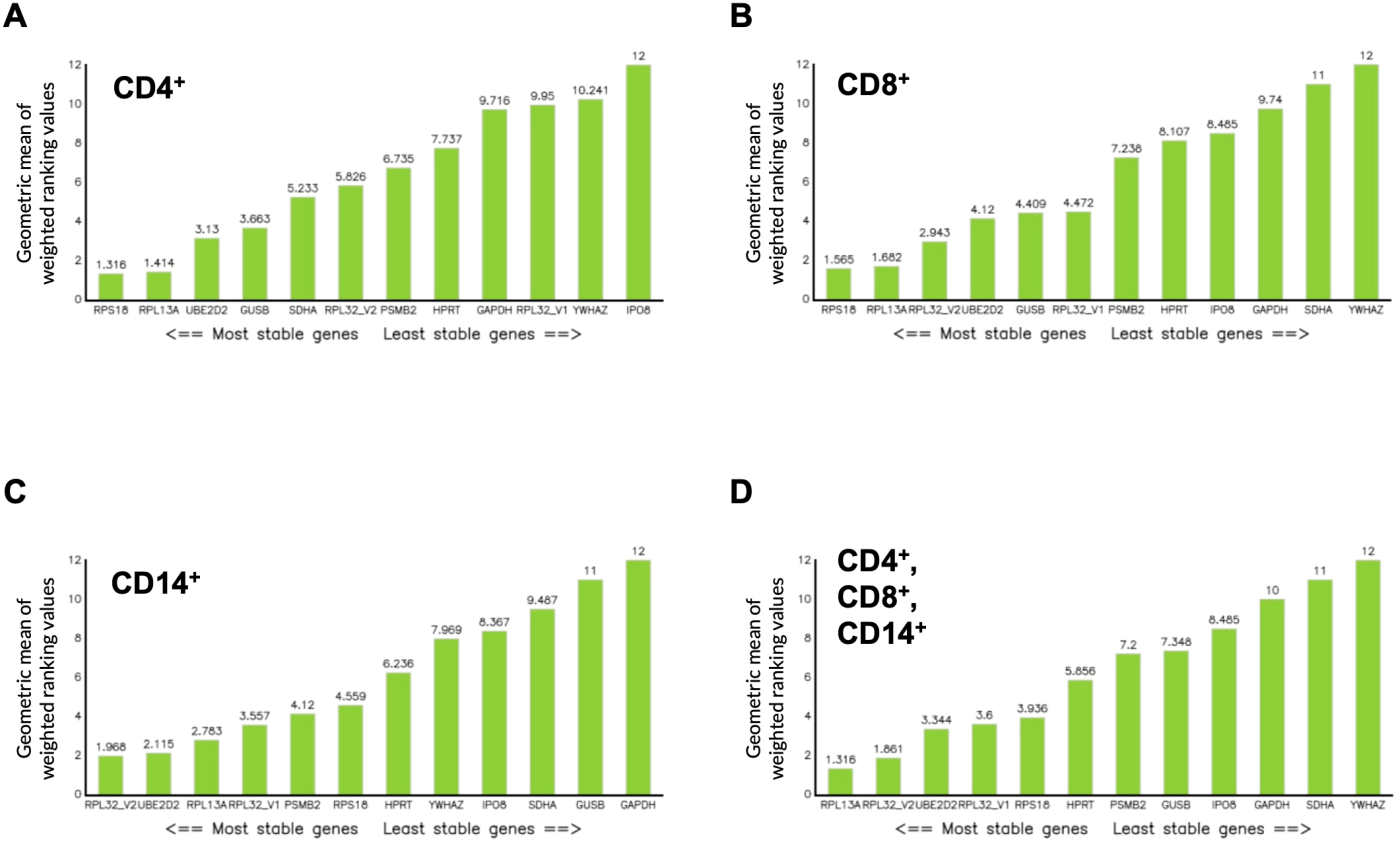
Ranking values of candidate reference gene evaluation in CD4^+^, CD8^+^, and CD14^+^ cells according to the RefFinder algorithm. Using C_q_ values of 12 candidate reference genes obtained under various stimulation conditions, the ranking algorithm RefFinder was applied to assess and rank gene expression stability in CD4^+^ cells **(A)**, CD8^+^ cells **(B)**, CD14^+^ cells **(C)**, and across all cell types combined **(D)**. RefFinder calculates a comprehensive stability ranking by computing the geometric mean of each gene’s weight across four distinct algorithms and subsequently re-ranking the candidate reference genes based on these geometric mean values. Genes with lower geometric mean values indicate higher expression stability and are shown on the left, whereas genes with higher geometric mean values indicate lower stability and are shown on the right.

To ensure reliable normalization of *STAT3* isoform expression, reference genes were systematically evaluated for stability across lymphocyte subsets and cytokine stimulation conditions. Twelve candidate reference genes (**Supplementary Table 1**) were assessed for expression stability across FACS-sorted healthy donor (HD) CD4^+^, CD8^+^ and CD14^+^ cells under unstimulated conditions and following 6 h stimulation with IFN-α, IL-6/sIL-6Rα or IL-10. C_q_ values across candidates spanned approximately 10.1 - 26.8, with substantial gene- and subset-specific differences (**Figures 2**–**4**). Several commonly used housekeeping genes (*GAPDH*, *GUSB*, *HPRT*) and other candidates (*RPL32*_V1, *PSMB2*, *YWHAZ*, *IPO8*, *SDHA*) displayed stimulus-dependent or inter-subset C_q_ variation exceeding pre-defined acceptance criteria (C_q_ window 16–22; intra-condition ΔC_q _≤1). Ribosomal genes *RPL13A*, *RPS18* and *RPL32*_V2 were stably expressed by ranking algorithms but exhibited C_q_ values substantially lower than *STAT3* targets, raising concern for ΔΔC_q_ bias in relative quantification. Algorithmic evaluation using RefFinder (geNorm, NormFinder, BestKeeper, comparative ΔC_q_) ranked different genes highest in specific subsets (**Supplementary Tables S2–S5**), yet when considering both stability and C_q_ proximity to *STAT3α* and *STAT3β*, *UBE2D2* demonstrated the most consistent performance across subsets and stimuli (**Figure 5**). Consequently, *UBE2D2* was selected as the normalization control for all *STAT3α*/STAT3*β* mRNA quantifications reported below.

### 3.3 Coordinated but cell-type-restricted mRNA induction of *STAT3* isoforms

To characterize isoform-specific transcriptional responses to cytokine signaling, *STAT3α* and *STAT3β* mRNA expression was quantified in FACS-purified CD4^+^, CD8^+^, CD14^+^ and CD19^+^ cells from HD buffy coats after 6 h stimulation with IFN-α, IL-6/sIL-6Rα or IL-10 (100 ng/ml each). Relative expression was calculated by the 2^−ΔΔCq^ method using *UBE2D2* as internal control. Biological replicates comprised samples from eight independent donors; donor numbers per subset and stimulus ranged from n = 3 - 7, with lower counts mainly due to limited CD19^+^ cell yield; exact n values are provided in the figure legends.

**Figure 6:**
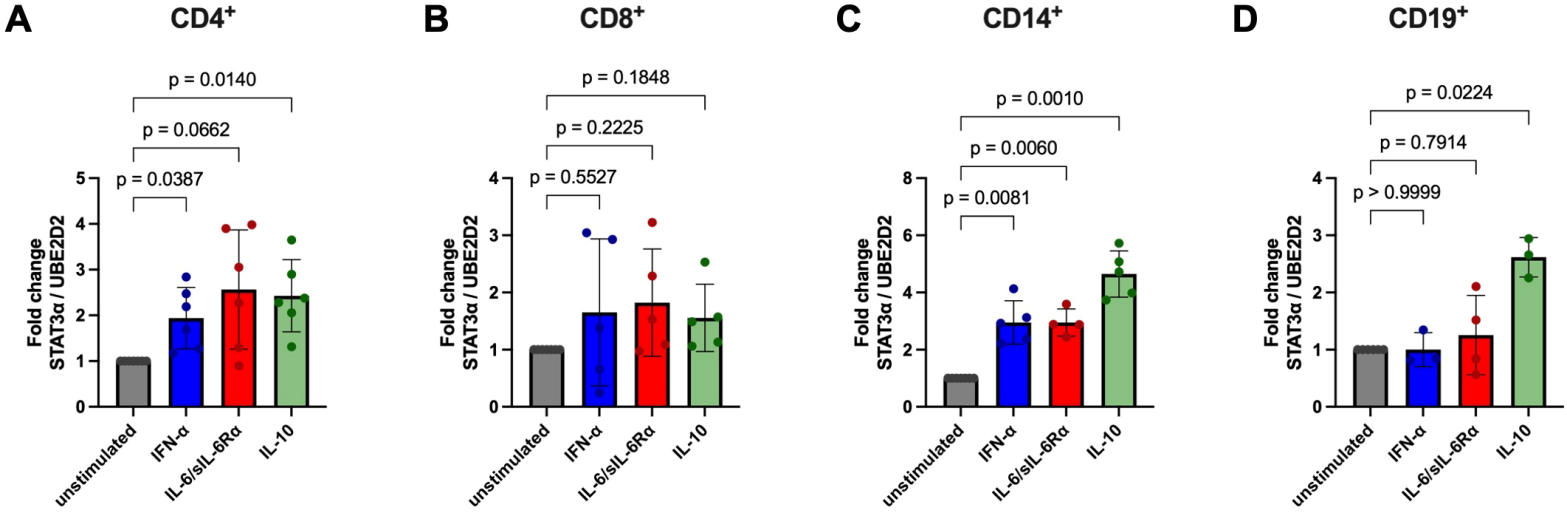
Analysis of *STAT3α* mRNA expression fold change in CD4^+^, CD8^+^, CD14^+^, and CD19^+^ cells after stimulation. CD4^+^ **(A)**, CD8^+^ **(B)**, CD14^+^ **(C)**, and CD19^+^ **(D)** cells were isolated from HD PBMCs by density gradient separation, sorted and stimulated without (grey symbols) or with IFN-α (100 ng/ml, blue symbols), IL-6/sIL-6Rα (100 ng/ml red symbols), or IL-10 (100 ng/ml, green symbols) for six hours. After RNA extraction and cDNA synthesis, C_q_ values of *STAT3α* and *UBED2* were determined *via* RT-qPCR. Fold change of *STAT3α* normalized to *UBED2* mRNA levels are shown as mean ± SD and individual values. Fold change differences between unstimulated and stimulated cells (paired for each donor) were tested for statistical significance by Two-way ANOVA with Tukey’s multiple comparisons test (p values are indicated). Donor numbers per condition (unstimulated, IFN-α, IL-6/sIL-6Rα, IL-10): CD4^+^ n = 7, 6, 6, 6; CD8^+^ n = 7, 5, 5, 5; CD14^+^ n = 7, 5, 4, 5; CD19^+^ n = 6, 3, 4, 3.

In CD4^+^ T cells, *STAT3α* increased by mean fold changes of 1.94 (IFN-α), 2.57 (IL-6/sIL-6Rα) and 2.43 (IL-10) relative to unstimulated controls (**Figure 6A**). CD8^+^ T cells showed a slight increase of *STAT3α* under the tested conditions without significant statistical differences (**Figure 6B**). CD14^+^ monocytes exhibited robust induction of approximately 2.95-fold after IFN-α or IL-6/sIL-6Rα and 4.65-fold after IL-10 stimulation (**Figure 6C**). CD19^+^ B cells displayed a modest but still statistically significant increase in *STAT3α* only with IL-10 (≈2.62-fold) (**Figure 6D**).

**Figure 7:**
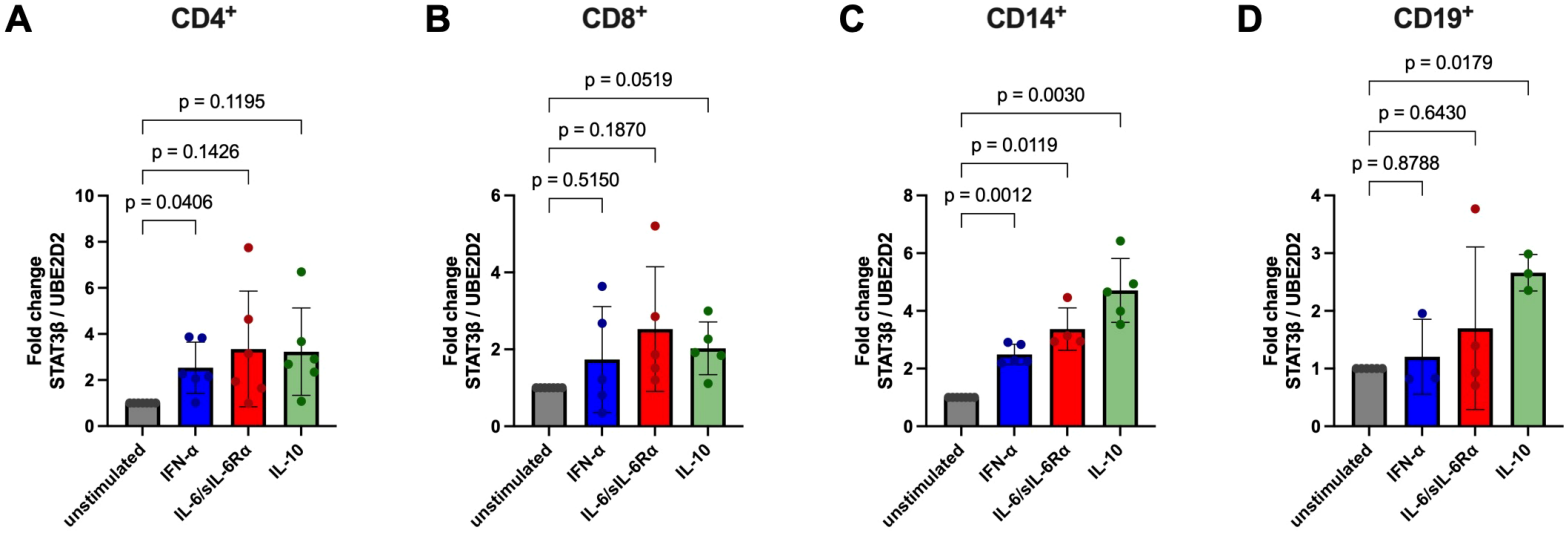
Analysis of *STAT3β* mRNA expression fold change in CD4^+^, CD8^+^, CD14^+^, and CD19^+^ cells after stimulation. CD4^+^ **(A)**, CD8^+^ **(B)**, CD14^+^ **(C)**, and CD19^+^ **(D)** cells were isolated from HD PBMCs by density gradient separation, sorted and stimulated without (grey symbols) or with IFN-α (100 ng/ml, blue symbols), IL-6/sIL-6Rα (100 ng/ml red symbols), or IL-10 (100 ng/ml, green symbols) for six hours. After RNA extraction and cDNA synthesis, C_q_ values of *STAT3β* and *UBED2* were determined *via* RT-qPCR. Fold change of *STAT3β* normalized to *UBED2* mRNA levels are shown as mean ± SD and individual values. Fold change differences between unstimulated and stimulated cells (paired for each donor) were tested for statistical significance by Two-way ANOVA with Tukey’s multiple comparisons test (p values are indicated). Donor numbers per condition (unstimulated, IFN-α, IL-6/sIL-6Rα, IL-10): CD4^+^ n = 7, 6, 6, 6; CD8^+^ n = 7, 5, 5, 5; CD14^+^ n = 7, 5, 4, 5; CD19^+^ n = 6, 3, 4, 3.

*STAT3β* expression generally paralleled *STAT3α* patterns with strong induction in CD4^+^ T cells after IFN-α (2.54-fold, **Figure 7A**) and in monocytes across all stimuli (up to 4.71-fold with IL-10, **Figure 7C**). In CD8^+^ cells, *STAT3β* changes were minor and did not reach statistical significance, similar to *STAT3α* (**Figure 7B**). CD19^+^ B cells showed a modest *STAT3β* increase only after IL-10 stimulation (≈2.66-fold, **Figure 7D**). Overall, mean fold changes of both isoforms were largely concordant across subsets, indicating parallel induction at the transcript level.

**Figure 8:**
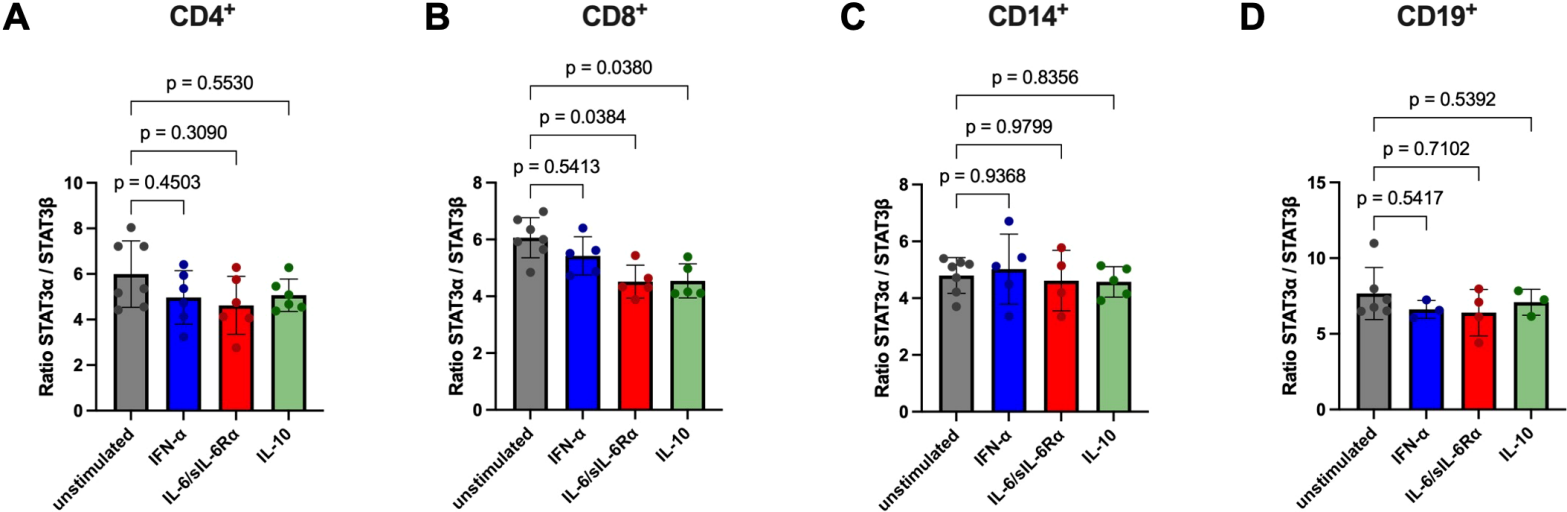
Analysis of *STAT3α*/*STAT3β* mRNA expression ratio in CD4^+^, CD8^+^, CD14^+^, and CD19^+^ after stimulation. CD4^+^ **(A)**, CD8^+^ **(B)**, CD14^+^ **(C)**, and CD19^+^ **(D)** cells were isolated from HD PBMCs by density gradient separation, sorted and stimulated without (grey symbols) or with IFN-α (100 ng/ml, blue symbols), IL-6/sIL-6Rα (100 ng/ml red symbols), or IL-10 (100 ng/ml, green symbols) for six hours. After RNA extraction and cDNA synthesis, C_q_ values of *STAT3α* and *STAT3β* were determined *via* RT-qPCR. Ratios of *STAT3α* normalized to *STAT3β* mRNA levels are shown as mean ± SD and individual values. Differences in *STAT3α/STAT3β* mRNA expression ratio between unstimulated and stimulated cells (paired for each donor) were tested for statistical significance by Two-way ANOVA with Tukey’s multiple comparisons test (p values are indicated). Donor numbers per condition (unstimulated, IFN-α, IL-6/sIL-6Rα, IL-10): CD4^+^ n = 7, 6, 6, 6; CD8^+^ n = 7, 5, 5, 5; CD14^+^ n = 7, 5, 4, 5; CD19^+^ n = 6, 3, 4, 3.

The ratio of *STAT3α* to *STAT3β* mRNA remained largely constant across cell types and stimuli. Baseline ratios (*STAT3α/STAT3β*) were approximately 5.99 in CD4^+^ cells and 6.06 in CD8^+^ cells (**Figures 8A, B**; across stimulation conditions these values varied only marginally (CD4^+^ range ~4.62 - 5.07; CD8^+^ range ~4.52 - 6.06). CD14^+^ monocytes showed ratios between 4.57 and 5.02 (**Figure 8C**), and CD19^+^ B cells were more α-biased (ratios ~6.40 - 7.67, **Figure 8D**). A small decrease in the STAT3*α/STAT3β* ratio was detected in CD8^+^ cells under IL-6/sIL-6Rα and IL-10 stimulation (ratio ≈4.52 - 4.55), while absolute isoform fold changes in this subgroup narrowly fell short of statistical significance individually. Overall, across all subsets and stimuli the mRNA α/β ratio centered near ~5.5 (≈85% *STAT3α*, 15% *STAT3β*).

Taken together, these results indicate coordinated induction of *STAT3α* and *STAT3β* transcripts across immune subsets, and that short-term cytokine stimulation substantially alters isoform abundance but, apart from limited subset-specific effects, does not appear to substantially reprogram the *STAT3α/STAT3β* splice ratio.

### 3.4 Predominance of STAT3α protein levels in CD4^+^ and CD8^+^ cells

To determine whether mRNA isoform patterns translate to the protein level, STAT3α and STAT3β protein abundance was assessed by semi-quantitative Western blot in HD CD4^+^ and CD8^+^ T cells after 8 h and 16 h stimulation with IFN-α or IL-6/sIL-6Rα (100 ng/ml). Protein-level analyses were focused on CD4^+^ and CD8^+^ T cells because these subsets provided sufficient material for robust biochemical quantification and represent primary lymphocyte populations in which IFN-α and IL-6–mediated STAT3 signaling is well characterized. The 8 h and 16 h time points were selected to allow sufficient time for cytokine-induced transcriptional changes to be translated into detectable protein abundance, consistent with prior studies assessing STAT3 or cytokine-responsive protein expression at similar intervals. Protein extracts were normalized for total protein, separated by SDS-PAGE, and immunoblotted for STAT3 isoforms and densitometric signals were normalized to an internal loading control.

**Figure 9:**
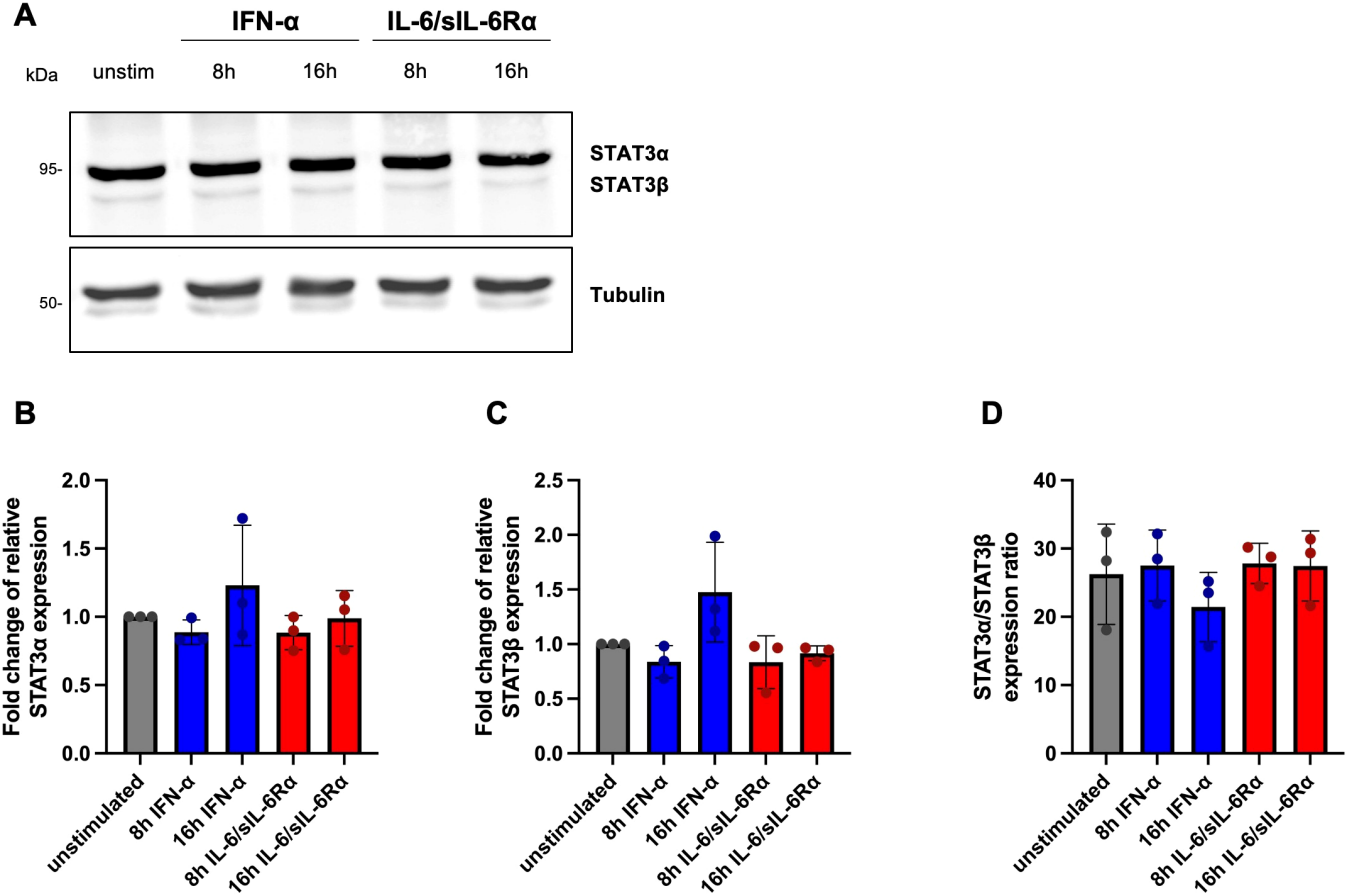
Ratio of STAT3α and STAT3β protein expression in CD4^+^ lymphocytes. **(A)** Representative Western blot analysis of total extracts from CD4^+^ PBMCs of HD unstimulated or stimulated with IFN-α or IL-6/sIL-6Rα for the indicated times. Blots were probed with an antibody against total STAT3. Tubulin was used as loading control. **(B)** Fold change of relative STAT3α protein expression, **(C)** fold change of STAT3β protein expression, and **(D)** STAT3α/STAT3β protein expression ratio in unstimulated (grey) and IFN-α (blue) or IL-6/sIL-6Rα (red) stimulated CD4^+^ cells. Differences were tested in at least three experiments for statistical significance by Two-way ANOVA with Tukey’s multiple comparisons test (p values are indicated).

In CD4^+^ T cells, protein expression of both isoforms remained stable across all conditions, and the STAT3α/STAT3β protein ratio showed only minor fluctuations (≈21–28), indicating consistent predominance of STAT3α (**Figure 9**). Under basal conditions, STAT3α accounted for 96.3% and STAT3β for approximately 3.7% of total STAT3 protein, corresponding to an approximately 26:1 α/β ratio, substantially higher than the transcript-level ratio (~5.5:1) (**Figure 8**).

**Figure 10:**
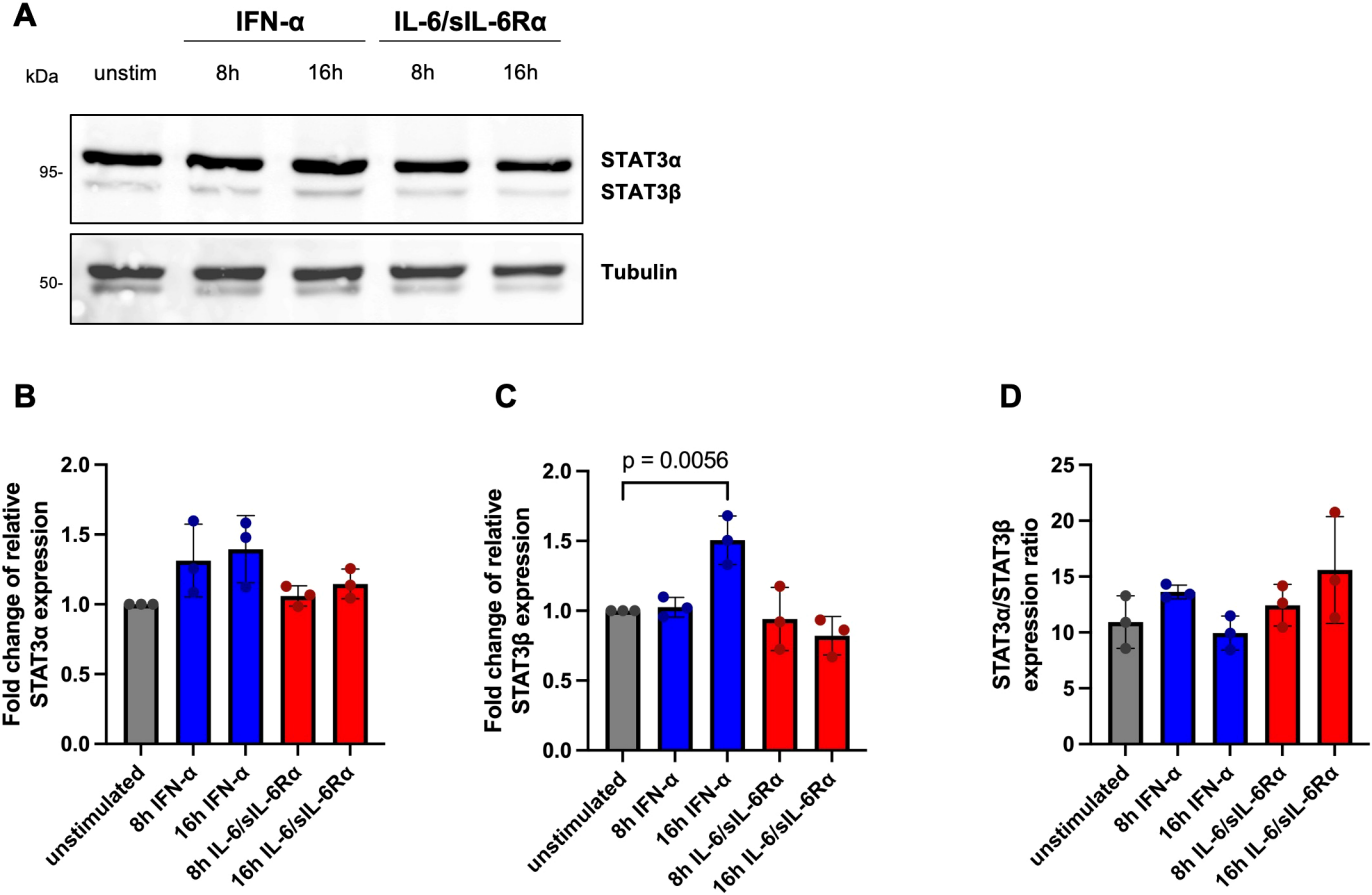
Ratio of STAT3α and STAT3β protein expression in CD8^+^ lymphocytes. **(A)** Representative Western blot analysis of total extracts from CD8^+^ PBMCs of HD unstimulated or stimulated with IFN-α or IL-6/sIL-6Rα for the indicated times. Blots were probed with an antibody against total STAT3. Tubulin was used as loading control. **(B)** Fold change of relative STAT3α protein expression, **(C)** fold change of STAT3β protein expression, and **(D)** STAT3α/STAT3β protein expression ratio in unstimulated (grey) and IFN-α (blue) or IL-6/sIL-6Rα (red) stimulated CD8^+^ cells. Differences were tested in at least three experiments for statistical significance by Two-way ANOVA with Tukey’s multiple comparisons test (p values are indicated).

In CD8^+^ T cells, STAT3α protein levels again showed no stimulus-dependent modulation, whereas STAT3β displayed a small but reproducible increase following 16 h IFN-α treatment (mean 1.51-fold, **Figure 10**). Protein STAT3α/STAT3β ratios in CD8^+^ cells were lower than in CD4^+^ cells (unstimulated ≈10.92), with measured values across conditions ranging from 9.95 to 15.59. In the unstimulated state, CD8^+^ cells displayed approximately 91.6% STAT3α and 8.4% STAT3β protein.

Overall, protein analyses confirm previous studies demonstrating STAT3α strongly predominates isoform balance at the protein level and that protein-level α/β ratios diverge markedly from mRNA ratios, suggesting post-transcriptional regulation that preserves STAT3α dominance despite stimulus-dependent transcriptional changes.

## 4 Discussion

In this study, we evaluated how IFN-α, IL-6/sIL-6Rα, or IL-10 as examples of canonical STAT3-activating cytokines affect STAT3α and STAT3β expression across primary human leukocyte subsets and assessed whether short-term stimulation alters isoform stoichiometry at the mRNA and protein level. Using validated isoform-specific qRT-PCR assays with empirically selected normalization (*UBE2D2*) and semi-quantitative Western blotting, we demonstrate three robust and interrelated observations: i) acute IFN-α, IL-6/sIL-6Rα and IL-10 stimulation increases total *STAT3* transcript abundance in a cell-type-dependent manner, ii) the *STAT3α*/*STAT3β* mRNA splice ratio is largely maintained (approx. 5.5 to 1) across subsets and stimuli, and iii) protein-level isoform stoichiometry is substantially skewed toward STAT3α, suggesting post-transcriptional mechanisms that preserve STAT3α predominance. Importantly, this study is primarily observational and quantitative in nature and is intended to establish a baseline framework for STAT3 isoform dynamics in primary human leukocytes rather than to resolve the underlying regulatory mechanisms.

Placing these results in context, prior studies have reported both stimulus-dependent modulation of *STAT3* gene expression (20, 21) and tissue-specific variation in α/β ratios (13–15, 22–24). The co-induction of both isoforms after stimulation supports a model of stimulus-responsive transcriptional control or autoregulation of STAT3 expression rather than isoform-selective transcriptional activation. Our finding of a largely preserved mRNA *STAT3α*/*STAT3β* ratio aligns with recent RNA-sequencing-based analyses showing that *STAT3α* is the predominant splice form in leukocytes (22), whereas the marked protein-level bias toward STAT3α is consistent with earlier biochemical reports of STAT3α dominance at steady state (6, 13, 14, 25, 26). The apparent transcript-protein decoupling extends these earlier observations by quantifying the degree of divergence across primary human T subsets and by showing that cytokine pulses largely preserve the splice architecture at the mRNA level while protein stoichiometry remains constrained. Thus, our data are consistent with, but do not directly demonstrate, a multilayered regulatory architecture in which alternative splicing sets a relatively stable transcript landscape that is subsequently modulated by translation efficiency, protein stability, and proteostatic pathways to determine steady-state isoform abundance. Accordingly, post-transcriptional or post-translational mechanisms discussed below should be interpreted as hypotheses that may explain the observed transcript–protein divergence.

Two related observations merit further consideration. First, in CD8^+^ T cells, IFN-α stimulation led to a modest but reproducible increase in detectable STAT3β protein. At the transcript level, this was accompanied by only a small, non-significant increase in *STAT3β* mRNA and a statistically significant but quantitatively limited reduction in the *STAT3α/STAT3β* mRNA ratio. Since comparable ratio shifts were not observed in other leukocyte subsets or stimulation conditions, this may hint at cell-type specific responsiveness. The magnitude of this ratio shift was small. Thus, in the context of otherwise parallel isoform induction, this CD8^+^-restricted observation represents a localized deviation that warrants further investigation to determine whether it reflects a reproducible cell type-specific regulatory effect. Second, the consistent divergence between mRNA and protein ratios across T cell subsets reinforces that transcript measurements alone do not reliably predict functional isoform composition. This divergence raises the possibility that post-transcriptional mechanisms may contribute to STAT3 isoform stoichiometry in defined cellular contexts, and therefore protein-level assessment is necessary to infer signaling capacity. One plausible hypothesis is that selective translational control or differential protein stability can transiently influence isoform balance in defined contexts. Potential explanatory mechanisms include differential ribosome recruitment, isoform-specific translation efficiency, modulation by RNA-binding proteins (e.g., PCBP1), selective microRNA-mediated regulation, or altered ubiquitin-proteasome-dependent turnover, mechanisms previously implicated in STAT3 regulation (16, 27–37). Future studies will be necessary to further elucidate such potential mechanisms. Collectively, these data suggest that processes beyond alternative splicing contribute to STAT3 isoform stoichiometry, although their relative contributions remain to be defined experimentally.

A limitation to this study was limited cell yields which constrained statistical power to detect small subset-specific shifts, particularly in CD19^+^cells. Western blotting was semi-quantitative and absolute protein copy numbers were not determined. Our analyses focused on acute (6–16 h) responses to up to three representative cytokines; longer stimulation, combinatorial signaling, additional cytokines relevant to B cell and myeloid biology (e.g., IL-21, G-CSF, TGF-β), or *in vivo* inflammatory milieus may result in different isoform dynamics. Functional readouts linking isoform stoichiometry to downstream transcriptional programs or cellular phenotypes were not performed but will be subject of follow-up studies. Considering these limitations, mechanistic conclusions regarding translation and degradation remain theoretical, as no ribosome profiling, targeted proteomics, ubiquitination assays, or analyses of specific splicing regulators were performed. Finally, *ex vivo* stimulation of peripheral blood cells may not fully recapitulate tissue microenvironments and *in vivo* inflammatory contexts.

Our observations establish a quantitative framework that warrants further mechanistic interrogation to resolve the functional physiology and implications of cell-type-specific STAT3 isoform ratios. First, extended temporal and cytokine conditions including IL-21, G-CSF, TGF-β and chronic inflammatory stimuli combined with larger donor cohorts will help distinguish transient from sustained isoform shifts and refine paired analyses. Second, applying absolute quantification approaches such as digital PCR for transcripts, targeted mass spectrometry or quantitative immunoassays for proteins may refine STAT3α/STAT3β stoichiometric estimates. Third, proteostasis-focused experiments, including turnover measurements and proteasome inhibition, may clarify whether differential degradation contributes to STAT3α predominance. Finally, investigation of candidate RNA-binding proteins or non-coding regulators, including factors such as PCBP1 or relevant miRNAs, could identify isoform-selective post-transcriptional interactions. Together, these approaches provide a concrete roadmap for converting the present descriptive findings into mechanistic insight. Importantly, assessing STAT3 isoform dynamics in cells from patients with AD-STAT3 HIES or following gene-editing interventions will further clarify how therapeutic manipulations affect isoform balance and function.

From a translational perspective, our findings have practical implications for (isoform-aware) therapeutic strategies targeting *STAT3*. Because both isoforms are co-induced by short-term cytokine signaling, yet downstream processing favors STAT3α at the protein level, therapeutic approaches that ablate STAT3β or replace endogenous STAT3 with a single isoform may disrupt physiologic post-transcriptional balance. Strategies that preserve endogenous splicing capacity or recapitulate physiological isoform ratios and allow post-transcriptional regulation may therefore better maintain normal STAT3 biology when correcting STAT3 defects, although these considerations remain to be validated experimentally.

In conclusion, acute cytokine exposure increases *STAT3* transcript abundance in a subset-specific manner without reprogramming the STAT3α/STAT3β splice ratio, while post-transcriptional and post-translational mechanisms might be relevant to a STAT3α predominance at protein level. As an observational and quantitative study, our work establishes a reference framework for STAT3 isoform dynamics in primary human immune cells. These findings highlight the importance of combined RNA and protein measurements to infer signaling competence and provide a foundation for future mechanistic studies aimed at defining how STAT3α/STAT3β isoform balance is regulated and how it can be leveraged in therapeutic contexts targeting STAT3 in immunodeficiency or inflammation.

## Data Availability

Raw Western Blot images have been published on Figshare: https://doi.org/10.6084/m9.figshare.31792942. Any inquiries regarding these contributions or further inquiries can be directed to the corresponding authors.
