# Supplementary material for: STAT3 isoform dynamics reveal robust splice ratio maintenance across cytokine-activated human immune cells
Source: Front Immunol. 2026 Apr 1;17:1792173. doi: 10.3389/fimmu.2026.1792173 (PMC13079131; doi:10.3389/fimmu.2026.1792173)
Supplement: Supplementary file 1 [file DataSheet1.docx]

Supplementary Material

1 Supplementary Tables

Table S1. Primer sequences of candidate reference genes used for qRT-PCR.

This table lists the forward and reverse primer sequences (5′→3′) for 11 potential reference genes evaluated in the qRT-PCR analyses. The included targets comprise *STAT3α*, *STAT3β*, *RPL32* (variants V1 and V2), *PSMB2*, *RPL13A*, *UBE2D2*, *YWHAZ*, *IPO8*, and *RPS18*. For each gene or isoform/variant, the corresponding primer pairs are provided with their full nucleotide sequences used for amplification of the respective target transcripts.

| **Primer name** | **Primer sequence (5′→3′)** |
| --- | --- |
| STAT3α/β_fw | TGACATTCCCAAGGAGGAGG |
| STAT3α_rv | ATTGCTGCAGGTCGTTGGTG |
| STAT3β_rv | TCCAAACTGCATCAATGAATGGTG |
| RPL32_V1_fw | ATCAGGCACCAGTCAGACCGAT |
| RPL32_V1_rv | GTTGCTCCCATAACCGATGTTGG |
| RPL32_V2_fw | GAAGTTCCTGGTCCACAACG |
| RPL32_V2_rv | GCGATCTCGGCACAGTAAG |
| PSMB2_fw | AGAGGGCAGTGGAACTCCTT |
| PSMB2_rv | AGGTTGGCAGATTCAGGATG |
| RPL13A_fw | CTCAAGGTGTTTGACGGCATCC |
| RPL13A_rv | TACTTCCAGCCAACCTCGTGAG |
| UBE2D2_fw | CTACGATCACAGTGGTCTCCAG |
| UBE2D2_rv | CGAGCAATCTCAGGCACTAAAGG |
| YWHAZ_fw | CCGTTACTTGGCTGAGGTTG |
| YWHAZ_rv | TTGCATTTCCTTTTTGCTGA |
| IPO8_fw | AGCTGGCTTGGCAGTTTATT |
| IPO8_rv | GCTGAATACGAGGCAGGAAT |
| RPS18_fw | GCAGAATCCACGCCAGTACAAG |
| RPS18_rv | GCTTGTTGTCCAGACCATTGGC |

Table S2. Ranking of candidate reference gene evaluation in CD4^+^, CD8^+^, and CD14^+^ cells combined.

Reference genes were evaluated with RefFinder, comparative ΔC_q_ method, BestKeeper, Normfinder, and geNorm algorithm. The genes positioned at the top show the highest expression stability, whereas the genes positioned at the bottom show the lowest expression stability according to the respective algorithm.

| **RefFinder** | | **Comparative ΔC_q_ method** | | **BestKeeper** | | **Normfinder** | | **geNorm** | |
| --- | --- | --- | --- | --- | --- | --- | --- | --- | --- |
| **Gene** | **Geom. mean** | **Gene** | **Std. dev.** | **Gene** | **Std. dev.** | **Gene** | **Stability value** | **Gene** | **Stability value** |
| RPL13A | 1.32 | RPL13A | 0.82 | UBE2D2 | 0.226 | RPL13A | 0.454 | RPL32_V2 \| RPL13A | 0.324 |
| RPL32_V2 | 1.86 | RPL32_V2 | 0.89 | RPL32_V2 | 0.376 | RPL32_V1 | 0.531 |  |  |
| UBE2D2 | 3.34 | RPL32_V1 | 0.89 | RPL13A | 0.414 | RPL32_V2 | 0.574 | RPS18 | 0.352 |
| RPL32_V1 | 3.6 | RPS18 | 0.9 | HPRT | 0.543 | RPS18 | 0.59 | RPL32_V1 | 0.447 |
| RPS18 | 3.94 | UBE2D2 | 0.96 | RPS18 | 0.577 | UBE2D2 | 0.67 | UBE2D2 | 0.529 |
| HPRT | 5.86 | GUSB | 1.02 | PSMB2 | 0.591 | GUSB | 0.734 | HPRT | 0.658 |
| PSMB2 | 7.2 | HPRT | 1.04 | RPL32_V1 | 0.603 | HPRT | 0.756 | PSMB2 | 0.726 |
| GUSB | 7.35 | PSMB2 | 1.07 | IPO8 | 0.897 | PSMB2 | 0.807 | IPO8 | 0.812 |
| IPO8 | 8.49 | IPO8 | 1.11 | GUSB | 0.999 | IPO8 | 0.842 | GUSB | 0.884 |
| GAPDH | 10 | GAPDH | 1.13 | GAPDH | 1.039 | GAPDH | 0.888 | GAPDH | 0.94 |
| SDHA | 11 | SDHA | 1.15 | SDHA | 1.087 | SDHA | 0.924 | SDHA | 0.981 |
| YWHAZ | 12 | YWHAZ | 1.17 | YWHAZ | 1.104 | YWHAZ | 0.933 | YWHAZ | 1.013 |

Table S3. Ranking of candidate reference gene evaluation in CD4^+^ cells.

Reference genes were evaluated with RefFinder, comparative ΔC_q_ method, BestKeeper, Normfinder, and geNorm algorithm. The genes positioned at the top show the highest expression stability, whereas the genes positioned at the bottom show the lowest expression stability according to the respective algorithm.

| **RefFinder** | | **Comparative ΔC_q_ method** | | **BestKeeper** | | **Normfinder** | | **geNorm** | |
| --- | --- | --- | --- | --- | --- | --- | --- | --- | --- |
| **Gene** | **Geom. mean** | **Gene** | **Std. dev.** | **Gene** | **Std. dev.** | **Gene** | **Stability value** | **Gene** | **Stability value** |
| RPS18 | 1.32 | RPS18 | 0.48 | RPL13A | 0.095 | RPS18 | 0.152 | RPL13A \| RPS18 | 0.194 |
| RPL13A | 1.41 | RPL13A | 0.49 | UBE2D2 | 0.138 | RPL13A | 0.187 |  |  |
| UBE2D2 | 3.13 | GUSB | 0.5 | RPS18 | 0.175 | GUSB | 0.192 | UBE2D2 | 0.218 |
| GUSB | 3.66 | UBE2D2 | 0.53 | RPL32_V2 | 0.235 | UBE2D2 | 0.278 | GUSB | 0.25 |
| SDHA | 5.23 | SDHA | 0.57 | GUSB | 0.244 | SDHA | 0.357 | SDHA | 0.286 |
| RPL32_V2 | 5.83 | RPL32_V2 | 0.63 | SDHA | 0.256 | PSMB2 | 0.467 | RPL32_V2 | 0.325 |
| PSMB2 | 6.74 | PSMB2 | 0.64 | PSMB2 | 0.341 | HPRT | 0.469 | PSMB2 | 0.385 |
| HPRT | 7.74 | HPRT | 0.65 | HPRT | 0.414 | RPL32_V2 | 0.473 | HPRT | 0.417 |
| GAPDH | 9.72 | GAPDH | 0.71 | RPL32_V1 | 0.429 | GAPDH | 0.525 | RPL32_V1 | 0.474 |
| RPL32_V1 | 9.95 | YWHAZ | 0.76 | YWHAZ | 0.52 | YWHAZ | 0.598 | GAPDH | 0.526 |
| YWHAZ | 10.24 | RPL32_V1 | 0.77 | GAPDH | 0.546 | RPL32_V1 | 0.632 | YWHAZ | 0.566 |
| IPO8 | 12 | IPO8 | 1.07 | IPO8 | 1.037 | IPO8 | 0.996 | IPO8 | 0.65 |

Table S4. Ranking of candidate reference gene evaluation in CD8^+^ cells.

Reference genes were evaluated with RefFinder, comparative ΔC_q_ method, BestKeeper, Normfinder, and geNorm algorithm. The genes positioned at the top show the highest expression stability, whereas the genes positioned at the bottom show the lowest expression stability according to the respective algorithm.

| **RefFinder** | | **Comparative ΔC_q_ method** | | **BestKeeper** | | **Normfinder** | | **geNorm** | |
| --- | --- | --- | --- | --- | --- | --- | --- | --- | --- |
| **Gene** | **Geom. mean** | **Gene** | **Std. dev.** | **Gene** | **Std. dev.** | **Gene** | **Stability value** | **Gene** | **Stability value** |
| RPS18 | 1.57 | RPS18 | 0.6 | RPL32_V2 | 0.209 | RPL13A | 0.342 | RPL13A \| RPS18 | 0.202 |
| RPL13A | 1.68 | RPL13A | 0.6 | UBE2D2 | 0.232 | RPS18 | 0.346 |  |  |
| RPL32_V2 | 2.94 | GUSB | 0.63 | RPS18 | 0.304 | GUSB | 0.354 | RPL32_V2 | 0.279 |
| UBE2D2 | 4.12 | RPL32_V1 | 0.64 | RPL13A | 0.338 | RPL32_V1 | 0.386 | UBE2D2 | 0.344 |
| GUSB | 4.41 | RPL32_V2 | 0.64 | RPL32_V1 | 0.367 | RPL32_V2 | 0.4 | RPL32_V1 | 0.383 |
| RPL32_V1 | 4.47 | UBE2D2 | 0.67 | HPRT | 0.478 | UBE2D2 | 0.436 | GUSB | 0.43 |
| PSMB2 | 7.24 | PSMB2 | 0.7 | GUSB | 0.509 | PSMB2 | 0.465 | PSMB2 | 0.494 |
| HPRT | 8.11 | IPO8 | 0.79 | PSMB2 | 0.528 | IPO8 | 0.594 | HPRT | 0.533 |
| IPO8 | 8.49 | HPRT | 0.79 | IPO8 | 0.568 | GAPDH | 0.626 | IPO8 | 0.593 |
| GAPDH | 9.74 | GAPDH | 0.81 | GAPDH | 0.598 | HPRT | 0.631 | GAPDH | 0.637 |
| SDHA | 11 | SDHA | 0.85 | SDHA | 0.684 | SDHA | 0.692 | SDHA | 0.672 |
| YWHAZ | 12 | YWHAZ | 1.01 | YWHAZ | 0.704 | YWHAZ | 0.887 | YWHAZ | 0.728 |

Table S5. Ranking of candidate reference gene evaluation in CD14^+^ cells.

Reference genes were evaluated with RefFinder, comparative ΔC_q_ method, BestKeeper, Normfinder, and geNorm algorithm. The genes positioned at the top show the highest expression stability, whereas the genes positioned at the bottom show the lowest expression stability according to the respective algorithm.

| **RefFinder** | | **Comparative ΔC_q_ method** | | **BestKeeper** | | **Normfinder** | | **geNorm** | |
| --- | --- | --- | --- | --- | --- | --- | --- | --- | --- |
| **Gene** | **Geom. mean** | **Gene** | **Std. dev.** | **Gene** | **Std. dev.** | **Gene** | **Stability value** | **Gene** | **Stability value** |
| RPL32_V2 | 1.97 | UBE2D2 | 0.8 | RPL32_V2 | 0.1066 | UBE2D2 | 0.45 | RPL32_V2 \| RPL13A | 0.272 |
| UBE2D2 | 2.11 | RPL32_V1 | 0.82 | RPL13A | 0.185 | PSMB2 | 0.477 |  |  |
| RPL13A | 2.78 | RPL32_V2 | 0.84 | RPS18 | 0.211 | HPRT | 0.492 | RPS18 | 0.298 |
| RPL32_V1 | 3.56 | PSMB2 | 0.85 | UBE2D2 | 0.238 | RPL32_V1 | 0.502 | RPL32_V1 | 0.346 |
| PSMB2 | 4.12 | RPL13A | 0.86 | RPL32_V1 | 0.283 | RPL32_V2 | 0.628 | UBE2D2 | 0.376 |
| RPS18 | 4.56 | RPS18 | 0.91 | PSMB2 | 0.293 | RPL13A | 0.642 | PSMB2 | 0.434 |
| HPRT | 6.24 | HPRT | 0.91 | IPO8 | 0.541 | YWHAZ | 0.688 | IPO8 | 0.503 |
| YWHAZ | 7.97 | YWHAZ | 1.01 | YWHAZ | 0.75 | RPS18 | 0.722 | HPRT | 0.628 |
| IPO8 | 8.37 | SDHA | 1.04 | HPRT | 0.767 | SDHA | 0.745 | YWHAZ | 0.723 |
| SDHA | 9.49 | IPO8 | 1.04 | SDHA | 0.885 | IPO8 | 0.847 | SDHA | 0.795 |
| GUSB | 11 | GUSB | 1.23 | GUSB | 1.215 | GUSB | 1.072 | GUSB | 0.887 |
| GAPDH | 12 | GAPDH | 1.44 | GAPDH | 1.455 | GAPDH | 1.347 | GAPDH | 0.979 |

2 Supplementary Figures

Figure S1. PCR screening for best T_annealing_ for isoform-specific amplification of *STAT3α* and *STAT3β* mRNA.
*STAT3α* and *STAT3β* specific forward and reverse primers were used in four PCR reactions with different DNA templates. Either genomic DNA (gDNA) of T cells, cDNA synthesized with (cDNA) or without a reverse transcriptase (-RT) from RNA of T cells or no DNA template (H_2_O) were used. PCR reactions were carried out with different annealing temperatures (56 °C, 58 °C, 60 °C, 62 °C, 64 °C, 66 °C, A-F). After the amplification DNA samples were run on a 2.5% agarose gel for 90 minutes. In unlabeled lanes, a 50 bp DNA ladder was loaded; in the 56 °C condition, a 1 kb DNA ladder was included in the rightmost lane.
